# Targeting lysophosphatidic acid receptor type 1 with Debio 0719 inhibits spontaneous metastasis dissemination of breast cancer cells independently of cell proliferation and angiogenesis

**DOI:** 10.3892/ijo.2011.1309

**Published:** 2011-12-20

**Authors:** MARION DAVID, JOHNNY RIBEIRO, FRANÇOISE DESCOTES, CLAIRE-MARIE SERRE, MARYSE BARBIER, MAXIMILIEN MURONE, PHILIPPE CLÉZARDIN, OLIVIER PEYRUCHAUD

**Affiliations:** 1 INSERM, UMR1033; 2 Université de Lyon; 3 Faculté de Médecine Lyon Est, Lyon; 4 Service de Biochimie Biologie Moléculaire Centre Hospitalier Lyon Sud, Hospices Civils de Lyon, Pierre Bénite, France; 5 Debiopharm S.A., Lausanne, Switzerland

**Keywords:** breast cancer, metastasis, lysophosphatidic acid, LPA_1_, Debio 0719

## Abstract

Metastasis is the main cause of death for cancer patients. Targeting factors that control metastasis formation is a major challenge for clinicians. Lysophosphatidic acid (LPA) is a bioactive phospholipid involved in cancer. LPA activates at least six independent G protein-coupled receptors (LPA_1–6_). Tumor cells frequently co-express multiple LPA receptors, puzzling the contribution of each one to cancer progression. All three receptors, LPA_1_, LPA_2_ and LPA_3_, act as oncogenes and prometastatic factors in the mouse mammary gland. The competitive inhibitor of LPA_1_ and LPA_3_ receptors, Ki16425, inhibits efficiently breast cancer bone metastases in animal models. We showed here that Debio 0719, which corresponds to the R-stereoisomer of Ki16425 exhibited highest antagonist activities at LPA_1_ (IC_50_=60 nM) and LPA_3_ (IC_50_=660 nM) than Ki16425 [IC_50_=130 nM (LPA_1_); IC_50_=2.3 μM (LPA_3_)]. *In vitro*, Debio 0719, inhibited LPA-dependent invasion of the 4T1 mouse mammary cancer cells. *In vivo*, early but not late administration of Debio 0719 (50 mg/kg p.o. twice daily) to BALB/c mice during the course of orthotopic 4T1 primary tumor growth reduced the number of spontaneously disseminated tumor cells to bone and lungs without affecting the growth of primary tumors and tumor-induced angiogenesis. We found that increased LPA_1_ mRNA expression in primary tumors of breast cancer patients correlated significantly with their positive lymph node status (p<0.001). Altogether, our results suggest that LPA_1_ controls early events of metastasis independently of cell proliferation and angiogenesis. Therefore, targeting this receptor with Debio 0719 has a high therapeutic potential against metastasis formation for breast cancer patients.

## Introduction

Lysophosphatidic acid (LPA) is a natural bioactive phospholipid with growth factor-like activities (1). LPA controls cell proliferation, motility and differentiation on many cell types including cancer cells due to the action of cell surface G protein-coupled receptors. At least six LPA receptors have been described as transducers of LPA activity (LPA_1–6_) (2). These six LPA receptors can be further subdivided into two groups, the EDG family of LPA receptors (including LPA_1–3_) and the purinergic family (including LPA_4–6_). LPA receptors are widely expressed in tissues. Among these receptors LPA_1_ has the most ubiquitous spectrum of expression in the organism (3). LPA is implicated in several physiological and pathological processes (immunological system, fertility, central nervous system, renal fibrosis, lung fibrosis, hair loss, cancer) (4). The more important source of LPA in the organism comes from platelet activity upon platelet aggregation (5). LPA can also be produced by cells, including tumor cells that express the nucleotide-pyrophosphate pyrophosphatase 2/autotaxin/ATX (6,7). Accumulating data over the past ten years indicate that LPA is involved in cancer progression (8). LPA might be involved in carcinogenesis since a series of studies reported that mRNA expression for LPA_2_ and LPA_3_ were elevated in numerous cancers (9–12). Recently, overexpression of each EDG family LPA receptor (LPA_1_, LPA_2_ and LPA_3_) in the mammary gland of MMTV-LPAr transgenic mice was shown to induce tumor formation and metastasis (13). We have shown that LPA-derived from platelets controls bone metastasis formation of breast cancer cells (14). Since its discovery as an autocrine motility factor produced by melanoma cells, ATX was shown to control the metastatic behavior of breast cancer cells (13,15). These data indicate that at least in the mammary gland LPA receptors might act as oncogenic drivers and that through the activation of these receptors, LPA might act as a pro-metastatic factor.

LPA_1_ mRNA was consistently found expressed in human primary breast tumors (11,14). We have shown previously that the LPA_1_ expressed by breast cancer cells controls bone metastasis formation in a mouse model (16). For that reason, the treatment of bone metastatic animals with the competitive inhibitor of LPA_1_ and LPA_3_ receptors, Ki16425 (17), inhibits efficiently the progression of bone metastases (16). However, because of the inoculation route of tumor cells directly into the blood stream, the role of LPA_1_ in spontaneous dissemination of breast cancer cells from a primary tumor site is still misunderstood. In light of these findings, we undertook the study of the role of LPA_1_ in the spontaneous dissemination of breast cancer cells to lungs and bone taking advantage of: i) the development of a new antagonist of LPA_1/3_ receptors Debio 0719, ii) the use of a mouse model exploiting the 4T1 mouse mammary cancer cell line which recapitulates the distinct steps of metastasis when engrafted into the mammary glands of syngenic BALB/C mice (18) and iii) a large collection of mRNA from primary tumors of breast cancer patients.

## Materials and methods

### Drugs and reagents

Lysophosphatidic acid (LPA, Oleoyl C18:1) and lysophosphatidylcholine (LPC) were obtained from Avanti Polar Lipids. Debio 0719 and Debio 0719-425 (S) which correspond to the R-stereoisomer and S-stereoisomer, respectively, of the competitive inhibitor of LPA_1_ and LPA_3_ receptors, Ki16425 (17), were synthesized by Debiopharm S.A.

### Cell culture

The 4T1 mouse mammary cancer cell line was obtained from the American Type Culture Collection and were cultured in complete media, DMEM medium (Invitrogen), 10% (v/v) fetal bovine serum (FBS, Perbio) and 1% penicillin/streptomycin (Invitrogen), at 37°C in a 5% CO_2_ incubator. 4T1 cells derive from a BALB/c spontaneous mammary carcinoma and are naturally resistant to 6-thioguanine (21). For disseminated 4T1 tumor cell (DTC) analysis, bone marrow cells were harvested from tibias and femurs of each animal by flushing. Cells were placed on 10-cm culture dishes in presence of complete media supplemented with 6-thioguanine 10 μM (Aldrich). After two weeks, resistant clones were fixed, stained and counted. The amount of rescued DTC was expressed in terms of cell clones/mm^2^.

### Calcium flux assays

Assays were conducted using Chemi-Screnn™ Calcium-optimized Chem-1 stable cell line expressing human recombinant LPA_1_ receptor (Milipore Corp., St. Charles, MO). To test for LPA_1_ agonist activity, cells were loaded with Fluo-4 NW and calcium flux in response to oleoyl-LPA, Ki16425 and Debio 0719 were determined in eight point 3-fold serial dilution dose starting at 10 μM. EC_80_ values for the reference agonist were determined upon agonist addition in a dose-dependent manner. IC_50_ values for Ki16425 and Debio 0719 were determined by addition of antagonist and incubation of 90 sec, followed by ligand stimulation at EC_80_ concentration.

### Patients and tumor characteristics

Studies involving human primary breast tumors were performed according to the principles embodied in the Declaration of Helsinki. Tissue biopsies were obtained as part of surgical treatments for the hormone receptor content determination. Remaining samples were included anony-mously in this study. All human experiments were approved by the Experimental Review Board from the Laennec School of Medicine that waived the need for consent. The study cohort corresponded to 104 pre-menopausal patients treated between October 1994 and October 2001 (37). Criteria for inclusion in the study were as follow: primary breast tumor without inflammatory features, no previous cancer therapy, and no identified metastasis at the time of diagnosis. Estrogen receptor (ER) and progesterone receptor (PgR) were assayed in cytosol using the radioligand reference method (EORTC, 1980). Results were expressed as fmol/mg cytosol protein. ER and PgR positive tumors contained >2 and >5 fmol/mg protein, respectively.

### RNA extraction

Breast cancer tissue biopsies were obtained by surgery, selected by the pathologist and immediately snap-frozen in liquid nitrogen until processing. The biopsies were pulverized using a ‘Mikro-Dismembrator’ (B. Braun Biotech International, Melsungen, Germany) and total RNAs were extracted using TRI Reagent (Sigma, St. Louis, MO). To remove any genomic DNA contamination, total RNAs were treated with RNAse-free DNAse I and purified using RNeasy micro-columns (Qiagen, Hilden, Germany). RNA quality was verified using an Agilent Bioanalyser 2100 (Agilent Technologies, Santa Clara, CA).

### Reverse transcription and quantitative real-time polymerase chain reaction (RT-QPCR)

Expression of LPA_1_ mRNA was quantified by real-time quantitative RT-PCR on an Eppendorf Mastercycler^®^ RealPlex (Invitrogen) using the SYBR^®^ Green PCR kit (Finnzymes). Quantifications were normalized to corresponding RNA L32 and TBP values. The cDNAs were amplified by PCR for 35 cycles with the following specific PCR primers: human LPA_1_, 5′-TGGCATTAAAAATTTTACAAAAACA-3′ (forward) and 5′-AATAGTTAACAACATGGGAATGG-3′ (reverse); human L32, 5′-CAAGGAGCTGGAAGTGCTGC-3′ (forward) and 5′-CAGCTCTTTCCACGATGGC-3′ (reverse); human TBP, 5′-TGGTGTGCACAGGAGCAAG-3′ (forward) and 5′-TTCACATCACAGCTCCCCAC-3′ (reverse). Each cycle consisted of 10 sec of denaturation at 95°C, 15 sec of annealing at 60°C for LPA_1_ and 67°C for L32 and TBP, followed by 10 sec of extension at 72°C. Experimental procedures were followed as described (15).

### Cell invasion assay

Invasion assays were carried out using Bio-Coat migration chambers (Becton-Dickinson) with 8-μm filters previously coated with Matrigel as described previously (38). 4T1 cells (2×10^5^) were plated in the upper chambers in presence of absence of increasing concentrations of Debio 0719 and LPA in the lower chambers in presence of 1% fetal bovine serum. After incubation for 24 h at 37°C in 5% CO_2_ incubator, cells that had migrated through the filters were fixed and stained. The membranes were mounted on glass slides, and cells from 10 random microscopic fields (magnification ×40) were counted. All experiments were run in duplicate, and invasion was expressed in terms of cells/mm^2^.

### Pharmacokinetic studies

Experiments were performed at Shanghai Medicilion Inc. (Shanghai, China) using CD-1 male mice. Debio 0719 was administered in a solution of 10% DMA, 5% Cremophor EL and 85% saline to mice. Debio 0719 was administered intravenously at a dose of 5 mg/kg as a 10 ml/kg bolus into the jugular vein. For oral exposure, Debio 0719 was administered via an oral gavage at a dose of 50 mg/kg in a volume of 20 ml/kg. Blood samples were collected from retro-orbital puncture in sodium heparin containing tubes (n=3 for each time-point). Plasma samples (100 μl) were transferred to Eppendorf tube, then 20 μl methanol and 500 μl internal solution (Lovastatin, 500 ng/ml) were added. After vortexing for 1 min and centrifuging for 5 min at 15,000 rpm, supernatant was transferred to new vials and 5 μl of plasma samples were analyzed for Debio 0719 concentration by liquid chromatography/mass spectrometry. The liquid chromatography was carried out using an Agilent liquid chromatograph (Agilent Technologies). Mass spectrometric analysis was performed using an API3000 (triple-quadrupole) instrument from ABI Inc (Concord, Ontorio, Canada) with an ESI interface. Data acquisition and control system were created using Analyst 1.4 software from ABI Inc.

### In vivo oncological studies

The mice used in our study were handled according to the rules of Décret no. 87–848 du 19/10/1987, Paris. The experimental protocol have been reviewed and approved by the Institutional Animal Care and Use Committee of the Université Claude Bernard Lyon-1 (Lyon, France). Studies were routinely inspected by the attending veterinarian to ensure continued compliance with the proposed protocols. BALB/C mice, 4 weeks of age, were housed under barrier conditions in laminar flow isolated hoods. Autoclaved water and mouse chow were provided *ad libitum*. Animals bearing tumor xenografts were carefully monitored for established signs of distress and discomfort and were humanely euthanized when these were confirmed. Tumor fad pad experiments were performed using 4T1 cells (10^5^ in 10 μl of PBS) injected into the fat pad of the 4th mammary gland of female BALB/c mice of 6 weeks of age (Charles River). Animals were treated *per os* with Debio 0719 (25 mg/kg twice daily or 50 mg/kg twice daily) from day 0 to 14 or from day 15 to 35 post tumor cell injection. Primary tumors were resected 14 days after tumor cell injection and tumor weights were measured. For spontaneous metastasis dissemination studies, 14 days after tumor cell injection, animals were anesthetized and primary tumors were surgically removed. Mice were then followed for an additional 3-week observation at which time they were sacrificed, lungs were collected for histological analysis and bone marrow cells were harvested for DTC quantification.

### Immunohistochemistry

Resected primary tumors were fixed and embedded in paraffin. Five μm sections were subjected to immunohistochemistry. Detection of the nuclear antigen Ki-67 was carried out as described previously (15). The Ki-67 mitotic index was calculated as the ratio of the number of Ki-67 positive nuclei to the total nucleus number per field and results were expressed as the percentage of Ki-67-positive nuclei. For microvessel detection, immunostaining was performed with a rabbit polyclonal antibody against von Willebrand factor (vWF), and a rat monoclonal antibody against mouse CD31 (PECAM-1). Tumor angiogenesis was evaluated using the Chalkley’s Grid. Data were expressed as the percentage of marks on the grid that cover stained vessels from 10 independent fields on each tumor tissue section.

### Statistical analysis

Data were analyzed with the StatView 5.0 software using unpaired Student’s t-test for *in vitro* and *in vivo* studies. Analysis of the distribution of LPA_1_ expression in relation to usual prognostic parameters was performed with the non-parametric Mann-Whitney test or Kruskall-Wallis test.

## Results

### Debio 0719 inhibits LPA/LPA_1_-stimulated calcium flux with a stronger potency than Ki16425

**Figure 1:**
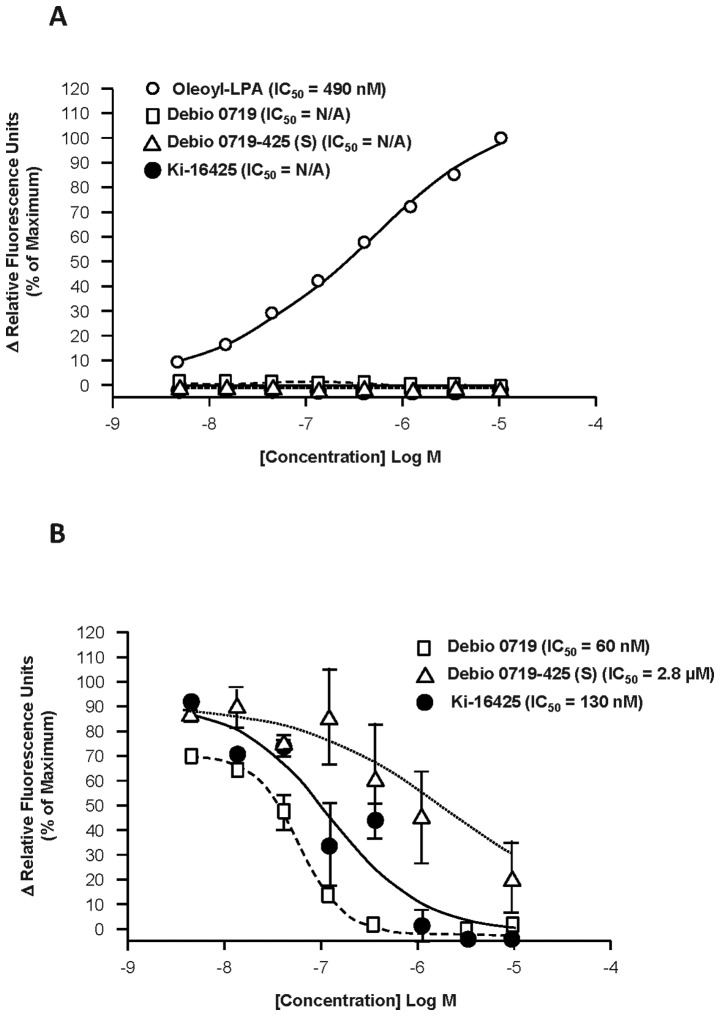
Calcium flux assay. (A) Assessment of Debio 0719-mediated agonism of the LPA_1_ receptor. Human LPA_1_-expressing Chem-1 stable cell line was incubated with increasing concentrations of Oleoyl LPA, Ki16425, Debio 0719 or Debio 0719-425(S) (0.0045–10 μM) and then assayed for stimulation of calcium flux. (B) Dose-dependent antagonism of LPA-induced calcium flux to human LPA_1_-expressing Chem-1 stable cell line was incubated with increasing concentrations of Ki16425, Debio 0719 or Debio 0719-425(S) (0.0045–10 μM) in the presence of 1 μM Oleoyl LPA.

Since its discovery, the competitive inhibitor Ki16425 was extensively used to address the role of LPA_1_ both *in vitro* and *in vivo* (16,17,19,20). To evaluate the role of LPA_1_ in metastasis, we first characterized the pharmaco-dynamic properties of two derivatives of Ki16425, Debio 0719 and Debio 0719-425(S). Debio 0719 and Debio 0719-425(S) correspond to the R-stereoisomer and S-stereoisomer, respectively, of the Ki16425, which is a racemic mixture of R- and S-stereoisomers, in a ratio of ~50:50. Debio 0719 and Debio 0719-425(S) were tested for agonist activity by incubating increasing concentrations of Debio 0719 and Debio 0719-425(S) (0.0045–10 μM) with Chem-1 cells expressing human LPA_1_ and measuring calcium flux (Fig. 1A). Like Ki16425, Debio 0719 and Debio 0719-425(S) showed no agonist activity at the LPA_1_ receptor at any concentration tested, whereas increasing concentrations of oleoyl LPA showed dose-dependent stimulation of calcium flux with an average EC_50_ of 490 nM. Debio 0719 inhibited LPA-induced calcium flux in LPA_1_-expressing Chem-1 cells in a dose-dependent manner, resulting in IC_50_ value of 60 nM (Fig. 1B). Parallel experiments showed that Ki16425 inhibited LPA-induced LPA_1_-dependent calcium flux in Chem-1 cells with a higher IC_50_ value of 130 nM and Debio 0719-425(S) with much higher IC_50_ value of 2.8 μM. Based on these results, Debio 0719 revealed 2-fold more potent than Ki16425 at inhibiting LPA_1_ cell signaling that control calcium flux. Thus, the R-stereoisomer can be considered as the active stereoisomer of Ki16425 to inhibit LPA/LPA_1_-induced calcium flux. Therefore, all subsequent experiments presented here were carried out by using only Debio 0719.

### Debio 0719 inhibits 4T1 breast cancer cell invasion in response to LPA

**Figure 2:**
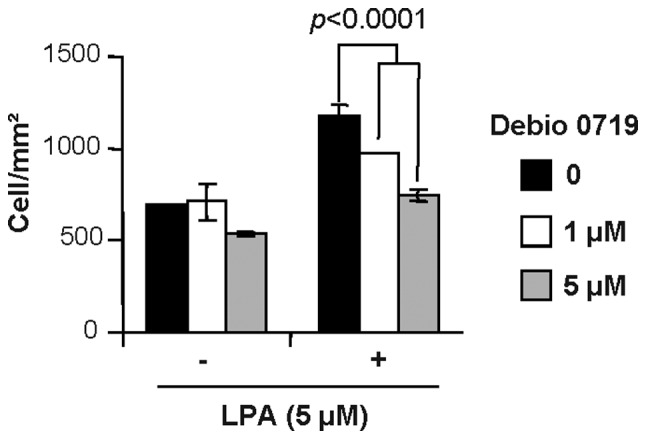
Debio 0719 inhibits LPA-induced 4T1 cell invasion by Debio 0719. Cell invasion was stimulated with LPA (5 μM) used as chemoattractant in presence of Debio 0719 (1 and 5 μM) placed in the upper chamber. Results are the mean ± SD of cells of 3 replicates and are representative of at least 3 independent experiments. Data are expressed as the number of cells/mm^2^.

Recent studies have shown that LPA_1_ is the main receptor that transduces the cell migratory activity of LPA (19). The 4T1 mouse mammary cancer cells mimic the successive steps of growth and metastasis of breast cancers observed in clinic when injected in the mammary fat-pad of immunocompetent BALB/c mice (18,21). These cells express all subtypes of LPA receptors including LPA_1_ (15). We found previously that 4T1 cells respond to LPA as a chemo-attractant in a cell invasion assay (15). We observed here that the migratory activity of LPA was dose-dependently blocked on cells treated with increasing concentrations of Debio 0719 (Fig. 2).

### Pharmacokinetics

**Figure 3:**
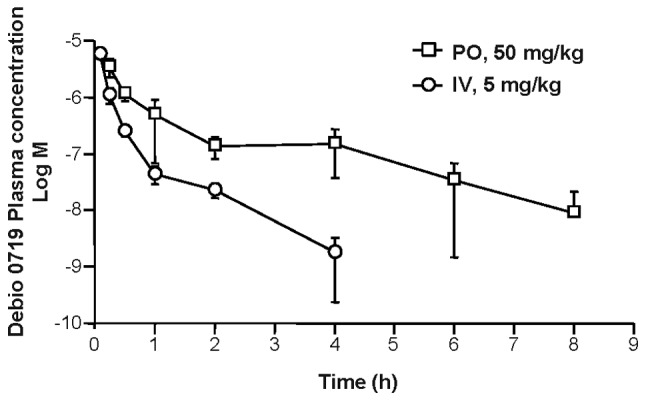
Pharmacokinetic profile of Debio 0719 *in vivo*. Debio 0719 plasma concentration curves for male CD-1 mice after a single oral (PO; 50 mg/kg) or intravenous (i.v., 5 mg/kg) administration of Debio 0719.

The therapeutic potential of a pharmacological compound is linked to its stability and bioavailability *in vivo*. Therefore, we next measured the plasma concentration time curves for Debio 0719 in male CD-1 mice after both intravenous and oral administration (Fig. 3). After oral dosing (50 mg/kg), Debio 0719 concentration peaked at 15 min with a C_max_ of 3.5 μM thereafter decreasing to ~10 nM by 8 h, yealding a *t1/2* of 0.98 h. After intravenous dosing (5 mg/kg) a C_max_ of 5.6 μM was observed within 5 min, which decreased to ~1 nM by 4 h, yealding a *t1/2* of 0.49 h. The oral exposure was high with an oral bioavailability 14.41. The detailed pharmacokinetic parameters for Debio 0719 are shown in Table I.

### Targeting LPA_1_ in vivo with Debio 0719 does not inhibit primary tumor growth of 4T1 cells

**Figure 4:**
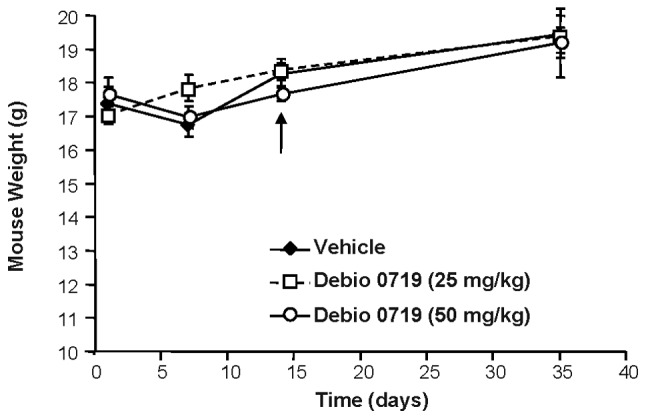
Effect of Debio 0719 treatment on the weight of 4T1-tumor bearing animals treated twice daily with Debio 0719 (25 and 50 mg/kg). Syngenic female Balb/C mice were inoculated with 4T1 cells by orthotopic injection into the mammary fat pad. Treatment with indicated doses of Debio 0719 started at day 0 and ended at day 15 post cell injection. Mouse weight was check at days 1, 7, 14, and 35. Arrow indicated the time of primary tumor resections.

**Figure 5:**
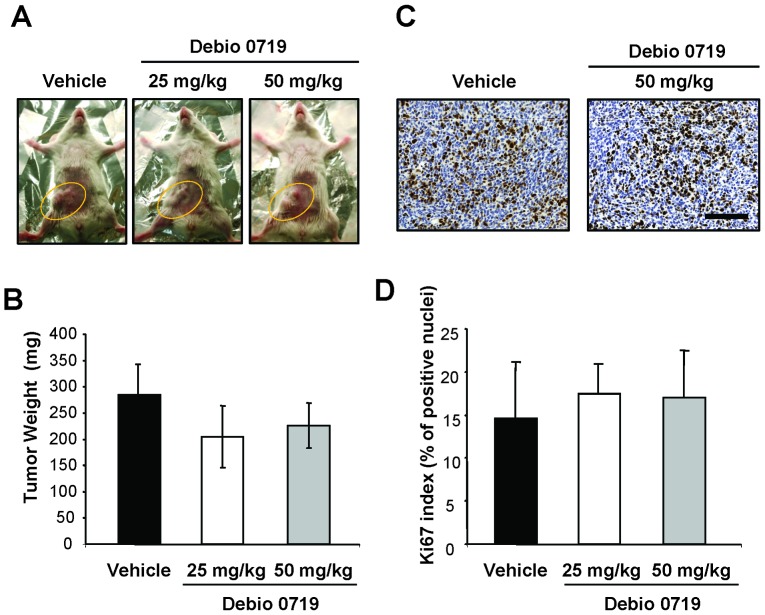
Effect of Debio 0719 treatment of female Balb/C mice on 4T1 primary tumor growth. 4T1 cells were injected in the mammary gland of normal syngenic female BALB/C mice. (A and B) Animals were treated *per os* twice daily with indicated doses of Debio 0719 from day 0 to 14. At day 14, primary tumors (A) (yellow circles) were resected and weighed (B) (results are expressed in mg as the mean ± SD). Primary tumors were embedded in paraffin. (C and D) Tumor tissue sections were analysed by immunohistochemistry using a specific antibody directed against the nuclear Ki-67 antigen. (D) The mitotic index was calculated as the percentage of nuclei positive for Ki-67. Results are the mean ± SD. Scale bar, 150 μm.

To analyze the role of LPA_1_ during the early steps of the metastatic dissemination of breast cancer cells, mice were inoculated orthotopically with 4T1 cells in the mammary gland and treated with Debio 0719 (25 and 50 mg/kg) administered orally *per os* twice daily, or with the vehicle only, from day 0 to 14 post cell injection. Treatments were stopped at day 14 at which time primary tumors were resected. We observed that a daily treatment of animals with Debio 0719 was well tolerated as judged by a constant gain of weight of the mice treated with Debio 0719 (25 and 50 mg/kg) twice daily as compared with mice treated with the vehicle (Fig. 4). Second, by measuring the weight of resected tumors, we found no difference in the burden of primary tumors between animals treated and not treated with Debio 0719 (Fig. 5A and B). This result was rather surprising based on our previous results showing a LPA_1_-dependent mitogenic activity of LPA on human MDA-B02 breast cancer cells *in vitro* and *in vivo* (14). By performing immunohistochemical analyses on 4T1 primary tumor sections, we found that the expression of the mitotic marker Ki-67 in the tumors was similar in animals treated or not treated with Debio 0719 (Fig. 5C and D). This result indicated that the treatment of mice with Debio 0719 had no effect on 4T1 cell proliferation *in vivo* at the site of implantation.

### Targeting LPA_1_ in vivo with Debio 0719 inhibits spontaneous metastasis of 4T1 cells to the lungs

**Figure 6:**
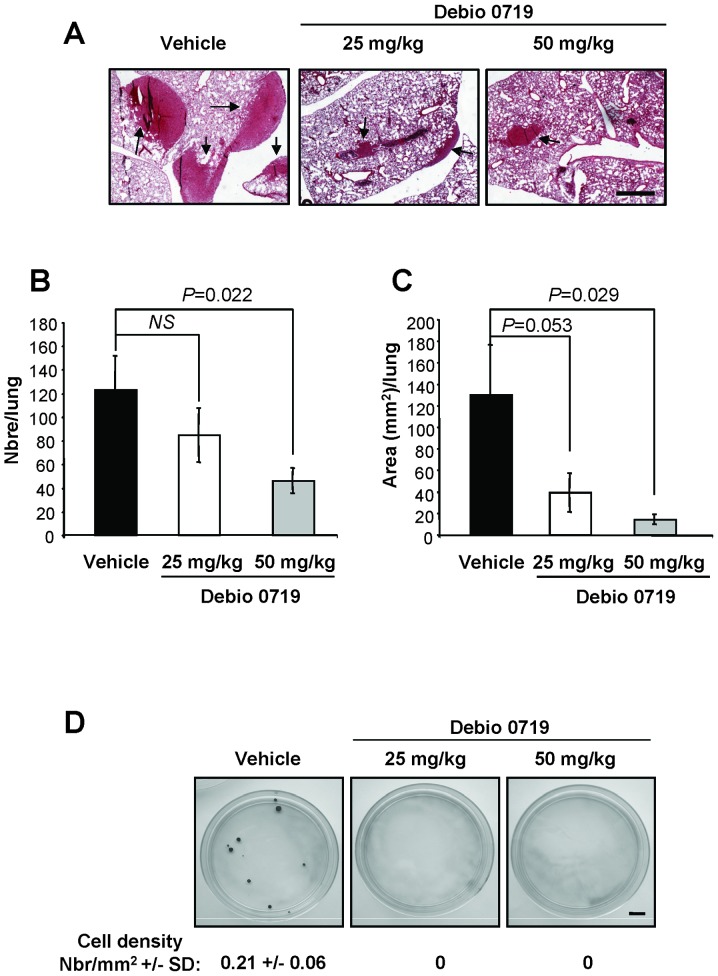
Effect of Debio 0719 treatment of female Balb/C mice on spontaneously metastasis dissemination of 4T1 cells to lungs (A-C) and bone (D). 4T1 cells were injected in the mammary gland of normal syngenic female BALB/C mice. Animals were treated *per os* twice daily with indicated doses of Debio 0719 from day 0 to 14. At day 14, primary tumors were resected. Animals were sacrificed 35 days after tumor cell injection and lungs (A) and bone marrow cells (D) were collected to quantify metastasis spreading of 4T1 cells. (A) Lung tissue sections stained with eosin. Arrows indicated metastatic foci. Scale bar, 200 μm. Quantification of the number (B) and the area (C) of lung metastasis foci were enumerated under microscope. Data were expressed as total number of foci/lung (B) and total foci area (in mm^2^)/lung (C) (means ± SD). (D) Bone marrow cells were harvested and plated on 10 cm culture dishes, in the presence of 6-thioguanine to rescue only 4T1-DTCs. 4T1-DTC clones were stained and counted. Data were expressed as the mean of cell density (in mm^2^) ± SD. Scale bar, 1 cm.

After primary tumor resections, animals treated with Debio 0719 from day 0 to 14 post cell injection were kept for an additional 21-day period and received no treatment until the day of sacrifice, where lungs were collected and the number and the area of metastatic foci were quantified by histological analysis. We observed that the number of lung metastasis foci was significantly reduced (inhibition = 62%, p=0.022) in animals treated with the higher dosing regimen of Debio 0719 compared to the untreated animals or to the group of mice receiving the lower dosing of Debio 0719 (Fig. 6A and B). Treatment of mice with the higher dosing of Debio 0719 (50 mg/kg twice daily) also decreased significantly the total area of lung metastases (inhibition = 89%, p=0.029) compared to untreated animals (Fig. 6C). Animals receiving the lower dosing of Debio 0719 presented also a reduction in the area of lung metastasis foci but the value did not reach statistical significance (p=0.053) (Fig. 6C).

### Targeting LPA_1_ in vivo with Debio 0719 inhibits dissemination of 4T1 cells (4T1-DTCs) to bone

We then asked whether the effect of Debio 0719 was restricted to the homing of 4T1 cells to the lungs or if its activity impaired the overall metastatic process. To address this question we analyzed the presence of 4T1-disseminated tumor cells (DTCs) at the bone site, as bone is one key target tissue of breast cancer derived metastases (22). At the time of sacrifice, bone marrow cells were harvested and 4T1-DTCs were rescued *in vitro* due to their endogenous resistance to the cytotoxic action of 6-thioguanine (21). At that day, we were not able to rescue any 4T1-DTCs from animals treated with either high or low dose of Debio 0719 (Fig. 6D).

**Figure 7:**
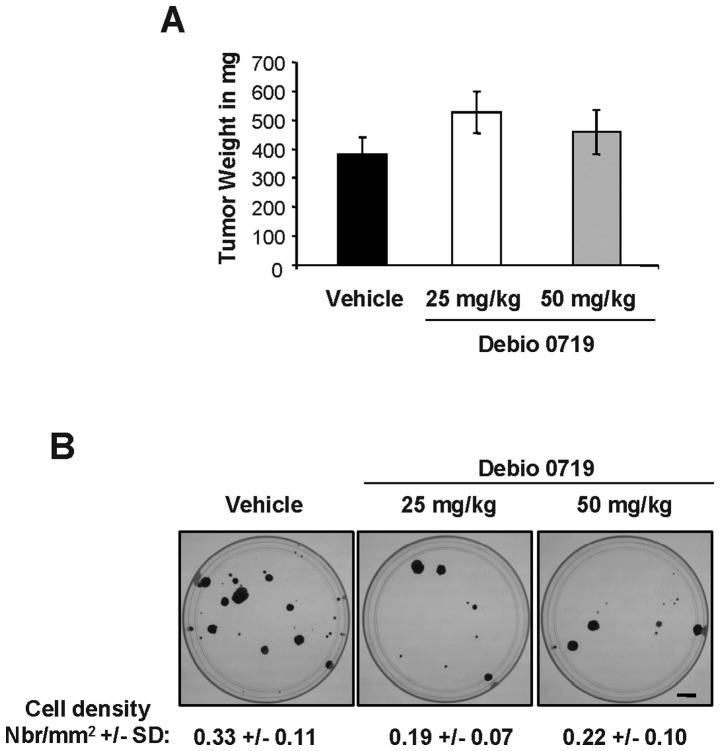
Effect of Debio 0719 treatment of female Balb/C mice on disseminated 4T1 tumor cell survival in bone. 4T1 cells were injected in the mammary gland of normal syngenic female BALB/C mice. (A) At day 14, primary tumors were resected and animals were randomized according to the tumor size. (B) Animals were treated *per os* twice daily with indicated doses of Debio 0719 from day 15 to 35. Animals were sacrificed 35 days after tumor cell injection and bone marrow cells were collected and plated on 10 cm cell culture dishes, in the presence of 6-thioguanine to rescue only 4T1-DTCs. 4T1-DTC clones were stained and counted. Data were expressed as the mean of cell density (in mm^2^) ± SD. Scale bar, 1 cm.

We then asked whether this observation could be due to an unexpected side effect of the compound on tumor cell survival in the bone marrow. We firstly generated 4T1 tumors in the mammary fat pad of BALB/c mice and then submitted the animals to the treatment with Debio 0719 in an adjuvant setting. Primary tumors were resected at day 14 and animals were randomized based on the size of resected tumors (Fig. 7A). Then, animals were treated or not with Debio 0719 (25 or 50 mg/kg) twice daily *per os*, for a 14 day period. At the time of sacrifice, we found no statistically significant difference in the number of 4T1-DTCs rescued from the bone marrow of animals treated with Debio 0719 compared to animals treated with the vehicle (Fig. 7B). These results indicated that the activity of Debio 0719 did not impair the survival of cancer cells already present in the bone marrow when the treatment was started. Altogether, the results suggested that the anti-metastatic activity of Debio 0719 was not restricted to a specific organ but instead affected the overall metastatic capacity of 4T1 cells.

### Targeting LPA_1_ in vivo with Debio 0719 inhibits spontaneous metastasis of 4T1 cells independently of tumor angiogenesis

**Figure 8:**
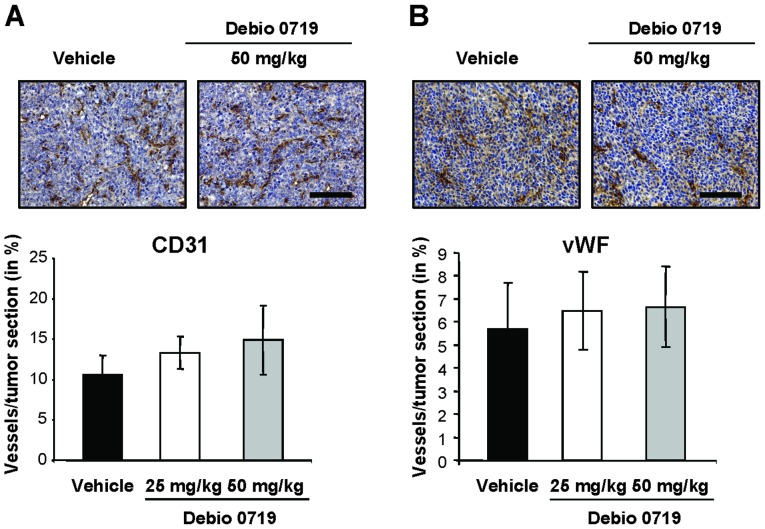
Effect of Debio 0719 treatment of female Balb/C mice on 4T1 tumor angiogenesis. Primary 4T1 tumors were collected as described in Fig. 5. Tumor tissue sections were analysed by immunohistochemistry using a specific antibody directed against CD31 (A) and vWF (B). The vessel density (in % of tissue section) was calculated using the Chalkley’s Grid. Results are the mean ± SD. Scale bar, 150 μm.

Angiogenesis is well known to control tumor growth and metastasis, and the LPA-LPA_1_ track might play a significant role in this process (23,24). By quantifying the levels of immunohisto-chemical endothelial cell markers (vWF and CD31) on 4T1 primary tumor sections, we found a similar level of angiogenesis in tumors collected from animals treated and untreated with Debio 0719 (Fig. 8). This result indicated that Debio 0719 did not significantly interfere with angiogenesis in the 4T1 mammary tumor model.

### High LPA_1_ expression at the primary tumor site links with positive lymph node status of pre-menopausal breast cancer patients

In clinic, lymph nodes are the first sites invaded by tumor cells during the metastasis process. Therefore, a high number of positive lymph nodes contributes to the poor prognosis for patients with breast cancers (25). We investigated whether LPA_1_ might be linked to lymph node invasion in breast cancer patients. We analyzed the expression levels of the mRNA for LPA_1_ in a series of 104 primary tumors from pre-menopausal patients without metastasis at the time of diagnosis. Higher LPA_1_ mRNA expression was significantly related to positive node tumors, p<0.001 (Table II). Any other classical prognostic factors (surgical tumor size, histological grade, ER and PgR status) revealed no difference (Table II). This suggested that LPA_1_ expression was associated with early steps of metastasis dissemination through invasion of lymph nodes of patients with breast cancers.

## Discussion

Metastasis is now considered as a very early event during tumor growth (26). We found previously that Ki16425, which is a competitive inhibitor of LPA_1_ and LPA_3_ receptors (17), inhibits efficiently the progression of breast cancer cell-mediated bone metastases in a mouse model (16). Here, we present pharmaco-kinetic and pharmacodynamic data for Debio 0719, which corresponds to the R-stereoisomer of Ki16425. Debio 0719 was evaluated for its ability to inhibit LPA_1_ activation by LPA. Inhibition of LPA_1_ activation was measured as a decrease in the LPA-stimulated calcium flux as Ki16425 was demonstrated to block LPA-induced elevation of intracellular calcium (17). The average IC_50_ values for the Debio- and Ki16425-mediated inhibition of LPA-stimulated human LPA_1_-expressing Chem-1 stable cell line was 60 and 130 nM, respectively, indicating that Debio 0719 was 2-fold more potent than Ki16425 at inhibiting LPA_1_-induced calcium flux. This was likely due to the presence of ~50% of the S-stereoisomer of Ki16425, which had a poor inhibitory activity on LPA/LPA_1_-stimulated calcium flux. It is noteworthy that Debio 0719 did not demonstrate any agonist effects on LPA_1_ at concentrations as high as 10 μM. Pharmacokinetic profile of Debio 0719 was assessed in male CD-1 mice. Debio 0719 was well tolerated after oral exposure with a half-life of 0.98 h. Administration of Debio 0719 to female Balb/C mice via an oral gavage at a dose up to 50 mg/kg twice daily for 14 days, did not induce noticeable deleterious effects. We observed that the treatment of mice with Debio 0719 during the early phase of tumor development inhibited efficiently the formation of spontaneous lung and bone metastases in a preclinical breast cancer mouse model exploiting the 4T1 mammary carcinoma cell line. In this model, the Debio 0719-mediated reduction in metastasis was not related to reduced angiogenesis at the primary tumor site, suggesting that LPA_1_ blockade interferes with another step of metastasis formation.

LPA is well known to have a mitogenic activity on a wild range of normal and cancer cell types (8). Recent reports demonstrated that LPA receptors, including LPA_1_ control directly the carcinogenesis through a pro-oncogenic action. By using an ErbB2/HER2 inducible system in the non-tumoral human MCF-10A breast cell line, Witt and colleagues found that LPA_1_ exhibits a fully oncogene activity in proliferation, migration and 3-dimentional acinar morphogenesis assays (27). However, a weak signal from ErbB2/HER2 revealed necessary for LPA_1_-induced cell action. An initial transformation step of MEF with c-myc and Tbx2 is also required for the oncogenic activity of LPA_1_, LPA_2_ and LPA_4_ (28). However, *in vivo*, individual over-expression of LPA_1_, as well as LPA_2_ and LPA_3_, driven by the MMTV promoter in transgenic mice leads to the formation of spontaneous mammary tumors within a year (13). Interestingly, over-expression of ATX, an LPA-producing enzyme, in MMTV-transgenic animals results in a similar spontaneous breast tumor formation (13). This work suggested that the sensitization of the breast epithelium to LPA, by increasing either the amount of cell surface LPA receptors or the local formation of LPA might prevail to initiate breast carcinogenesis rather than the control of carcinogenesis by a unique subtype of LPA receptor. We demonstrated recently that modulating the local production of LPA at the primary site of 4T1 breast tumors in animals, through stable down-regulation of ATX using an shRNAi strategy, decreases spontaneously lung metastasis formation but has no impact on primary tumor growth (15). Here, we found no effect on the growth of 4T1 mammary tumors in animal treated with Debio 0719. These results suggest that targeting LPA or its receptor LPA_1_ in breast cancer patients might most likely not lead to successful inhibition of primary tumor growth. This hypothesis is supported by the absence of link between the size or grade, and the levels of LPA_1_ expression among breast tumors analyzed throughout the present study. It could therefore be postulated that clinical trials with compounds targeting LPA signaling in breast cancer patients should rely on innovative clinical end-points, instead of RECIST criteria. Recently, a monoclonal antibody to RANKL (Denosumab) was approved to treat bone metastasis based on clinical end-points such as time to skeletal related events, rather than standard criteria (29).

LPA receptors share many intracellular signaling pathways that control cell behavior (30). LPA is known to induce migration and invasion of breast cancer cells through the mobilization of LPA receptors and downstream activation of the β-arrestin/Ral signaling pathway (31,32). Among LPA receptors, LPA_1_ was identified as the transducer of the migration activity of LPA on neoplastic and non-neoplastic cells (19). However, the idea of having one LPA receptor devoted to one function is probably not realistic. Overexpression of LPA receptors (LPA_1_, LPA_2_, LPA_3_) individually in the mammary gland of MMTV-transgenic mice induces the formation of distant metastases in up to 45% of tumor bearing animals (13). However, among LPA receptors, LPA_1_ might play a key role in the metastasis process of breast cancers. We found that high expression of LPA_1_ distributed with the nodal status of pre-menopausal breast cancer patients suggesting that increased expression of this receptor might contribute to early steps of breast cancer cell metastasis. It was demonstrated, since its discovery as a metastasis suppressor gene (33) that *Nm23-H1* down-regulates LPA_1_ expression in breast cancers (34). LPA_1_ re-introduction in breast cancer cells expressing *Nm23-H1* was sufficient to rescue these cells from inhibited migration and to induce metastasis formation *in vivo* (35,36).

In conclusion, by using an immunocompetent mouse model, based on orthotopic implantation of breast cancer cells, we found that blocking LPA_1_ activity *in vivo* with Debio 0719 during the early phase of tumor growth inhibited efficiently bone and lung metastasis formation. This anti-metastatic effect of Debio 0719 was mainly due to inhibition of cell invasion but not of cell proliferation and angiogenesis. Altogether, our results suggest that the level of LPA_1_ expression at the site of primary tumors might control very early events during the metastasis process of breast cancers and that targeting LPA_1_ with Debio 0719 has a high therapeutic potential against metastasis formation for breast cancer patients.

## Figures and Tables

**Table I:** Pharmacokinetic parameters for Debio 0719 in male CD-1 mice.

Dose route	Intravenous (n=3)	Oral (n=3)
Dose (mg/kg)	5	50
Plasma clearance (l/h/kg)	6.66	
T_1/2_ (h)	0.49	0.98
Bioavailability (% EF)		14.41
C_max_ (μg/ml)	2960	1658
Time to maximum concentration (h)	0.08	0.25

All values represent the mean for each parameter.

**Table II:** Distribution of LPA_1_ mRNA expression in different subsets of cases defined by the usual prognostic factors.

Prognostic factors	n	Median	P-value
Surgical tumor size			
<20 mm	37	5.19	
≥20 mm	64	5.51	0.607
Histological type			
Ductal	89	5.46	
Lobular	12	5.00	0.793
Histological grade^a^			
GI	12	5.36	
GII	43	5.46	
GIII	29	5.00	0.960
Node status			
Neg	47	3.24	
Pos	57	6.68	<0.001
ER status			
Neg	27	5.46	
Pos	77	5.37	0.556
PgR status			
Neg	23	5.55	
Pos	81	5.25	0.935
ER and/or PgR			
Neg	34	5.56	
ER and PgR			
Pos	70	5.04	0.584

a Histological grade defined only in ductal carcinoma.

P-values correspond to Mann-Whitney test or Kruskall Wallis test (histological grade).
